# Atazanavir/ritonavir monotherapy as maintenance strategy in HIV-1 treated subjects with viral suppression: 96-week analysis results of the MODAT study

**DOI:** 10.7448/IAS.17.4.19806

**Published:** 2014-11-02

**Authors:** Vincenzo Spagnuolo, Laura Galli, Alba Bigoloni, Silvia Nozza, Antonella d'Arminio Monforte, Andrea Antinori, Antonio Di Biagio, Stefano Rusconi, Giovanni Guaraldi, Simona Di Giambenedetto, Adriano Lazzarin, Antonella Castagna

**Affiliations:** 1 Department of Infectious Diseases, IRCCS San Raffaele Hospital, Milan, Italy; 2 Clinic of Infectious and Tropical Diseases, S Paolo Hospital, University of Milan, Milan, Italy; 3 Clinical Department, National Institute for Infectious Diseases IRCCS Lazzaro Spallanzani, Rome, Italy; 4 Division of Infectious Diseases, Azienda Ospedaliera San Martino, Genoa, Italy; 5 Division of Infectious Diseases, Ospedale Luigi Sacco, University of Milan, Milan, Italy; 6 Department of Medical and Surgical Sciences, University of Modena and Reggio Emilia, Modena, Italy; 7 Institute of Clinical Infectious Diseases, Catholic University of the Sacred Heart, Rome, Italy

## Abstract

**Introduction:**

The 48-week interim analysis of the MODAT study showed that confirmed virologic failure (CVF) was more frequent in patients simplifying to ATV/r monotherapy compared to maintaining ATV/r-based triple therapy. The DSMB recommended stopping study enrollment but continuing follow-up of enrolled patients. We present the 96-week efficacy analysis.

**Material and Methods:**

Multicentre, randomized, open-label, non-inferiority trial (non-inferiority margin −10%). Treatment failure (TF) was defined as CVF (two consecutive HIV-RNA >50 cp/mL) or discontinuation for any cause. In the monotherapy arm, patients with CVF re-introduced their previous NRTIs and remained in the study if HIV-RNA <50 copies/mL within 12 weeks of re-intensification.

**Results:**

101 patients evaluated (Figure 1): 85% males, 21% HCV-positive, median (IQR) age of 42 (36–48) years, baseline CD4+ 576 (447–743) cells/µL. In the 96-week analysis (ITT; TF=failure), efficacy was 64% (32/50) in the monotherapy arm and 63% (32/51) in the triple-therapy arm (difference +1.3%, 95% CI −17.5–20.1). Fourteen patients in monotherapy and two in triple-therapy arm had CVF; median HIV-RNA was 136 (72–376) copies/mL. In monotherapy arm, no PI or NRTI associated resistance mutations were observed at CVF. All patients who re-intensified re-suppressed. In monotherapy arm, TF was more frequent in HCV-co-infected patients (64% vs 28%; p=0.041). In the secondary analysis (ITT; re-intensification=success), 82% (41/50) in monotherapy arm and 63% (32/51) in triple-therapy arm were on study at week 96 (difference +19.3%, 95% CI 2.2–36.3). SAEs occurred in four (8%) patients in the monotherapy arm (one left basal pneumonia, one acute coronary stenosis, one traumatic lesion, one nephrolithiasis) and two (4%) in the triple therapy arm (one sepsis, one renal failure). Drug-related adverse events (AEs) leading to discontinuation were three (6%) in the monotherapy arm (two AEs occurred in patients after successful re-intensification) and 12 (23.5%) in the triple-therapy (p=0.023).

**Conclusions:**

Despite the small sample size, the primary 96-week analysis showed that simplification to ATV/r monotherapy showed inferior efficacy to maintaining ATV/r triple-therapy but appeared to be superior when re-intensification was considered success.

**Figure 1:**
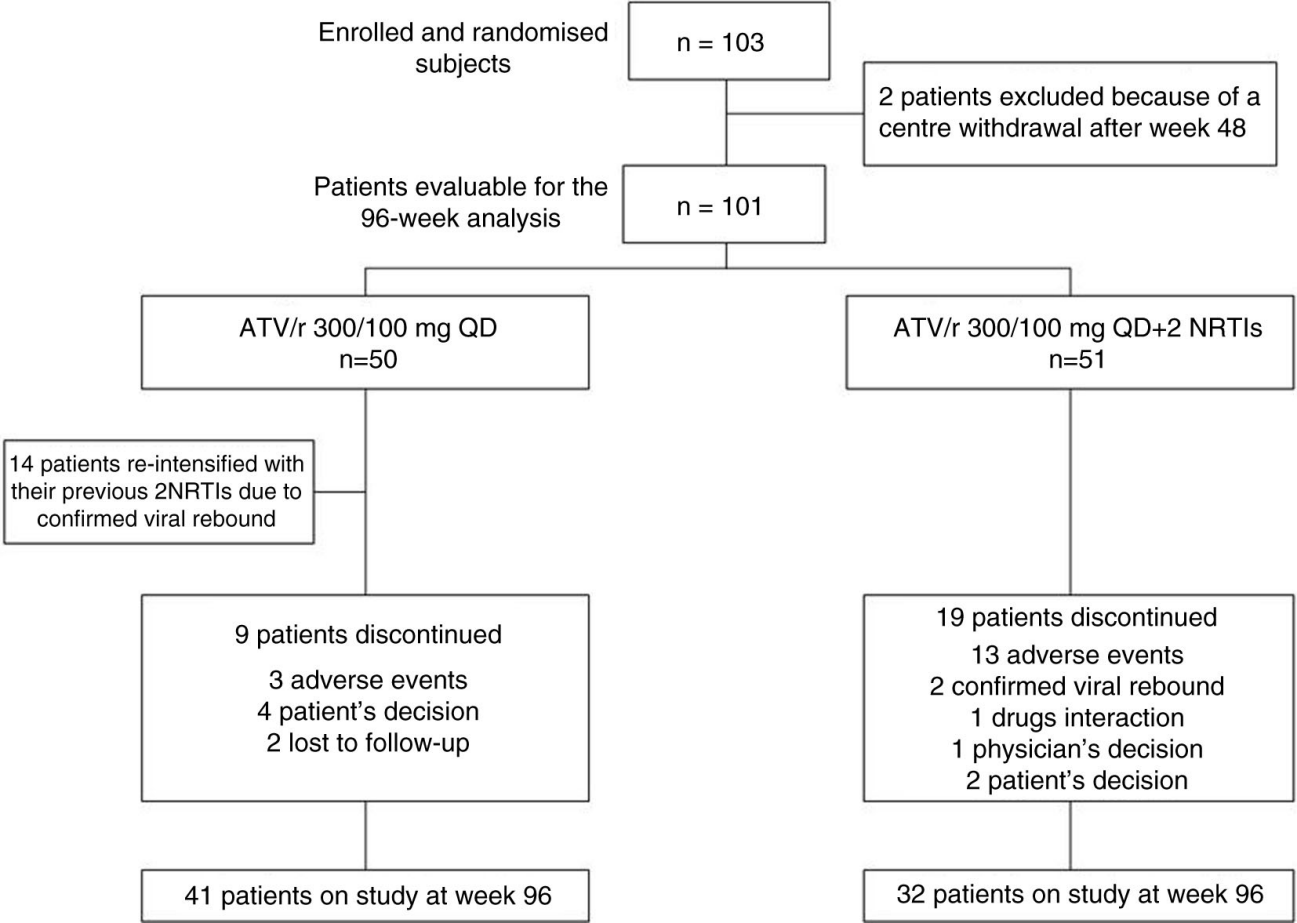
The MODAT trial: 96-week patients disposition.

